# Ischemic colitis presenting as a colonic mass: a case report and diagnostic challenges

**DOI:** 10.3389/fmed.2024.1503190

**Published:** 2025-01-08

**Authors:** Lin Xu, Yuqi Wu, Shangjin Li, Xinbo Chen, Dong Zhang, Boqian Chen, Shaoju Guo

**Affiliations:** ^1^Institute of Gastroenterology, Shenzhen Traditional Chinese Medicine Hospital, The Fourth Clinical Medical College of Guangzhou University of Chinese Medicine, Guangzhou, China; ^2^Guangzhou University of Chinese Medicine, Guangzhou, China

**Keywords:** ischemic colitis, colon mass, endoscopic findings, personalized treatment, case report

## Abstract

Ischemic colitis (IC) is a multifaceted condition that often manifests with nonspecific symptoms such as abdominal pain and bloody diarrhea, particularly in older adults with vascular risk factors. Diagnosis is supported by elevated levels of white blood cells, lactate, and C-reactive protein (CRP). Computed tomography (CT) imaging typically reveals wall thickening and fat stranding in watershed areas. Colonoscopy may demonstrate mucosal erythema, ulceration, or necrosis. IC can be differentiated from inflammatory bowel disease (IBD), diverticulitis, and colorectal cancer based on symptom patterns and imaging findings. The absence of specific biomarkers can complicate diagnosis, potentially causing delays. Illustrating these challenges is the case of a 53-year-old male patient who arrived at the hospital exhibiting abdominal pain and diarrhea. Enhanced CT scans and colonoscopy identified a mass in the ileocecal region of the colon, and subsequent tissue biopsy revealed ischemic lesions in the submucosa. Initially diagnosed with IC, the patient’s symptoms gradually improved with conservative treatment, which included antibiotics, fluid resuscitation, and bowel rest. Follow-up endoscopy showed significant lesion improvement, and no recurrence was detected during subsequent follow-ups. This case illustrates the healing process of IC as manifested by colon mass under endoscopy. Also, it highlights the critical importance of timely diagnosis and personalized treatment strategies in atypical presentations to improve patient outcomes.

## Introduction

Ischemic colitis (IC) is a condition caused by reduced blood flow to the intestines, which can be life-threatening (1, 2). It presents with a range of symptoms, including abdominal pain, hematochezia, and diarrhea. Diagnosing IC is particularly difficult due to the lack of specific clinical and biological markers. Endoscopic examination is crucial for diagnosing IC (3), but its findings often need to be distinguished from other gastrointestinal disorders such as colorectal tumors and inflammatory bowel disease, complicating the diagnostic process. Therefore, clinicians must remain highly vigilant and consider a broad differential diagnosis. We report a case of IC that appeared as a colonic mass in the ileocecal region during endoscopy. After symptomatic treatment, which included enhancing microcirculation and administering anti-infective therapy, significant improvement in the intestinal wall was observed on colonoscopy 4 weeks later. This article highlights an atypical presentation of IC as a colon mass on endoscopy. By providing this clinical reference, we aim to alert medical professionals to exercise caution with similar cases, thus preventing misdiagnosis and treatment delays.

## Case presentation

**Figure 1:**
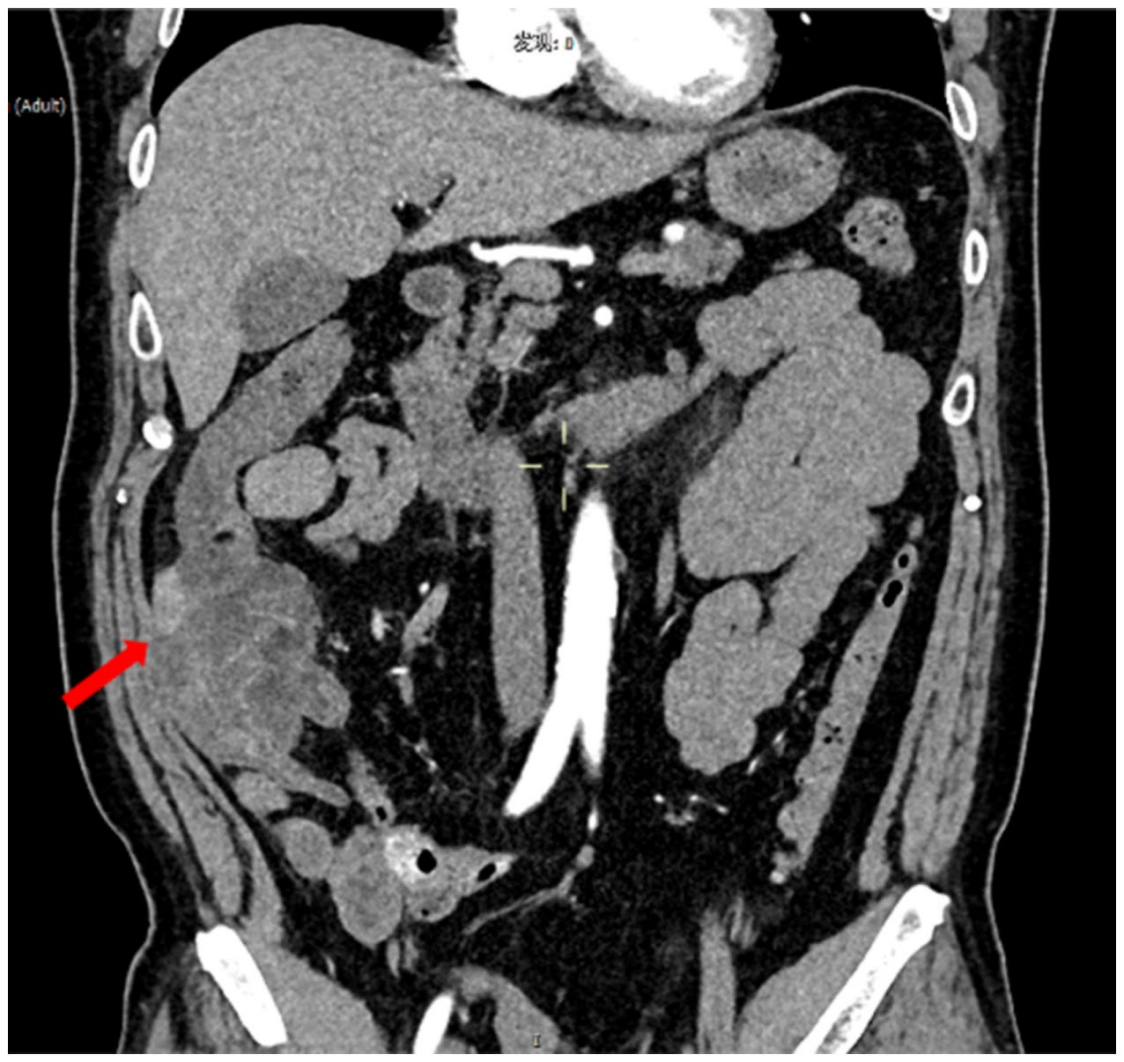
Images of abdominal CT. CT scans of the abdomen indicated significant thickening in the lower segment of the ascending colon and the ileocecal region, with intestinal shadows observed within. Enhanced imaging revealed multiple ring signs, and a slightly enhanced nodular shadow measuring approximately 24 mm was identified within the intestinal tract. Additionally, the surrounding fat gaps exhibited vague oozing.

A 53-year-old male presented with abdominal pain that began 2 days prior, characterized as intermittent and non-localized, accompanied by over ten episodes of watery diarrhea. He did not report melena, mucus, or pus in the stools and experienced one episode of vomiting gastric contents without coffee-ground material. Due to persistent symptoms, he sought emergency care. Examination revealed red, loose stools with significant red blood (++++) and 5–8 white blood cells per high power field (HPF). Hemoglobin and transferrin tests were positive. Blood tests showed a white blood cell count of 9.68 × 10^9/L, neutrophil count of 7.58 × 10^9/L, neutrophil percentage of 78.4%, and lymphocyte percentage of 16.5%. Renal function, liver function, pancreatic enzymes, myocardial enzymes, electrolytes, and coagulation tests were normal. Abdominal ultrasound indicated aortic plaques but no abnormalities in the abdominal aorta or renal arteries. CT scans of the abdomen revealed significant thickening and a mass-like appearance in the distal ascending colon, suggestive of a tumor or tumor-associated intussusception, warranting further examination (Figure 1). The right upper abdominal mesenteric fat space appeared obscured, indicating potential mesenteric fat inflammation.

The patient’s medical history includes hypertension, diabetes mellitus, atrial fibrillation, coronary artery disease, hyperlipidemia, and hyperuricemia. His long-standing medication regimen includes valsartan for blood pressure, rivaroxaban for anticoagulation, extended-release metoprolol for heart rate control, atorvastatin for lipid management, febuxostat for uric acid reduction, and insulin aspart 30 for blood glucose control. Due to persistent symptoms, he was admitted for further management.

**Figure 2:**
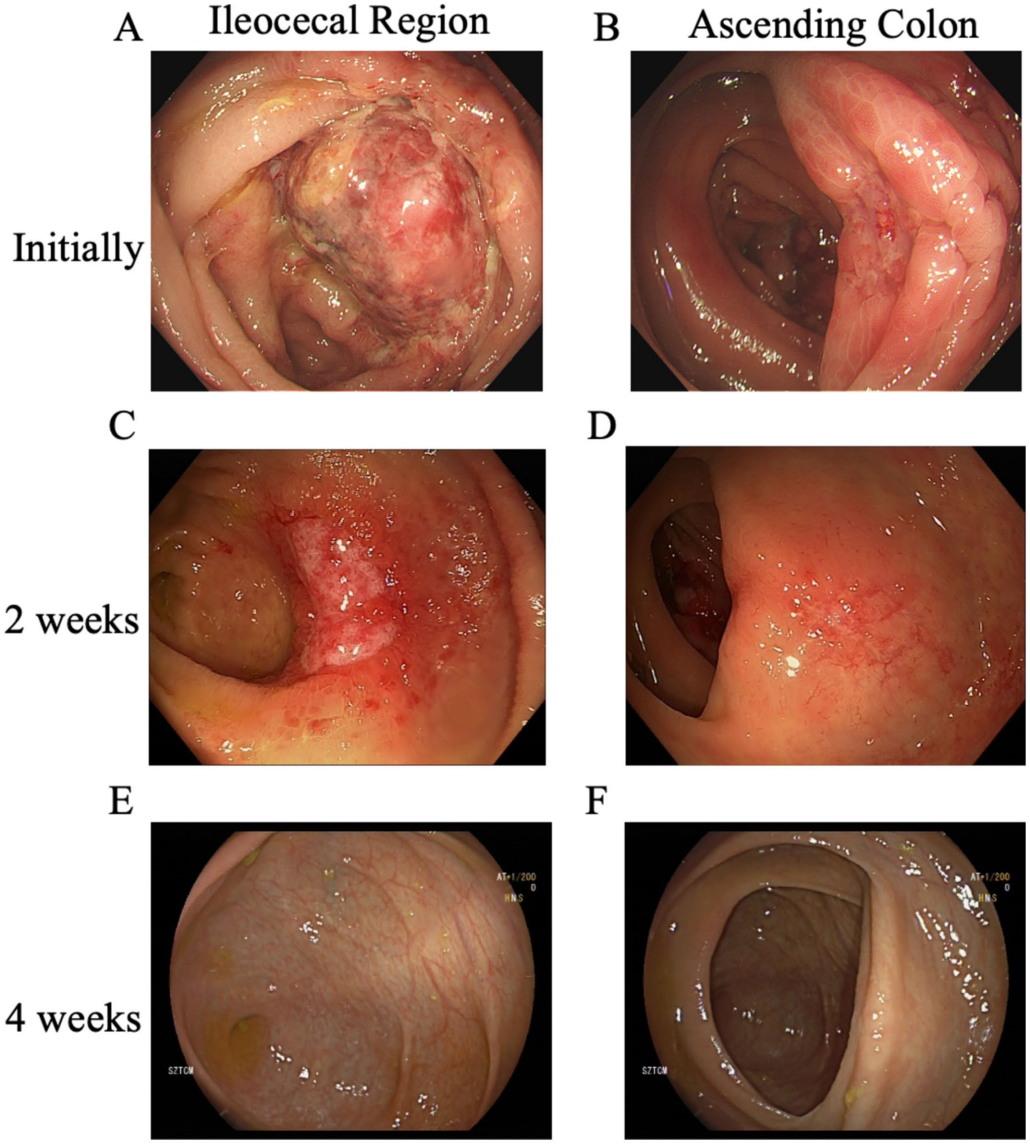
Evolution of lesions in the ileocecal and ascending colon across three colonoscopies. **(A)** The initial colonoscopy identified a mass-like lesion in the ileocecal region, and **(B)** patchy erosion with shallow ulcer formation was observed near the ileocecal area of the ascending colon. Two weeks later, **(C)** a follow-up colonoscopy showed that the mass in the ileocecal region had evolved into a depressed ulcer, while **(D)** the original ulcer in the ascending colon had become a shallow erosion. Four weeks after the initial examination, **(E)** a subsequent colonoscopy revealed that the entire colon had normalized, with no ulcers, erosions, or other lesions observed in the ileocecal region, and **(F)** the ascending colon was also clear.

**Figure 3:**
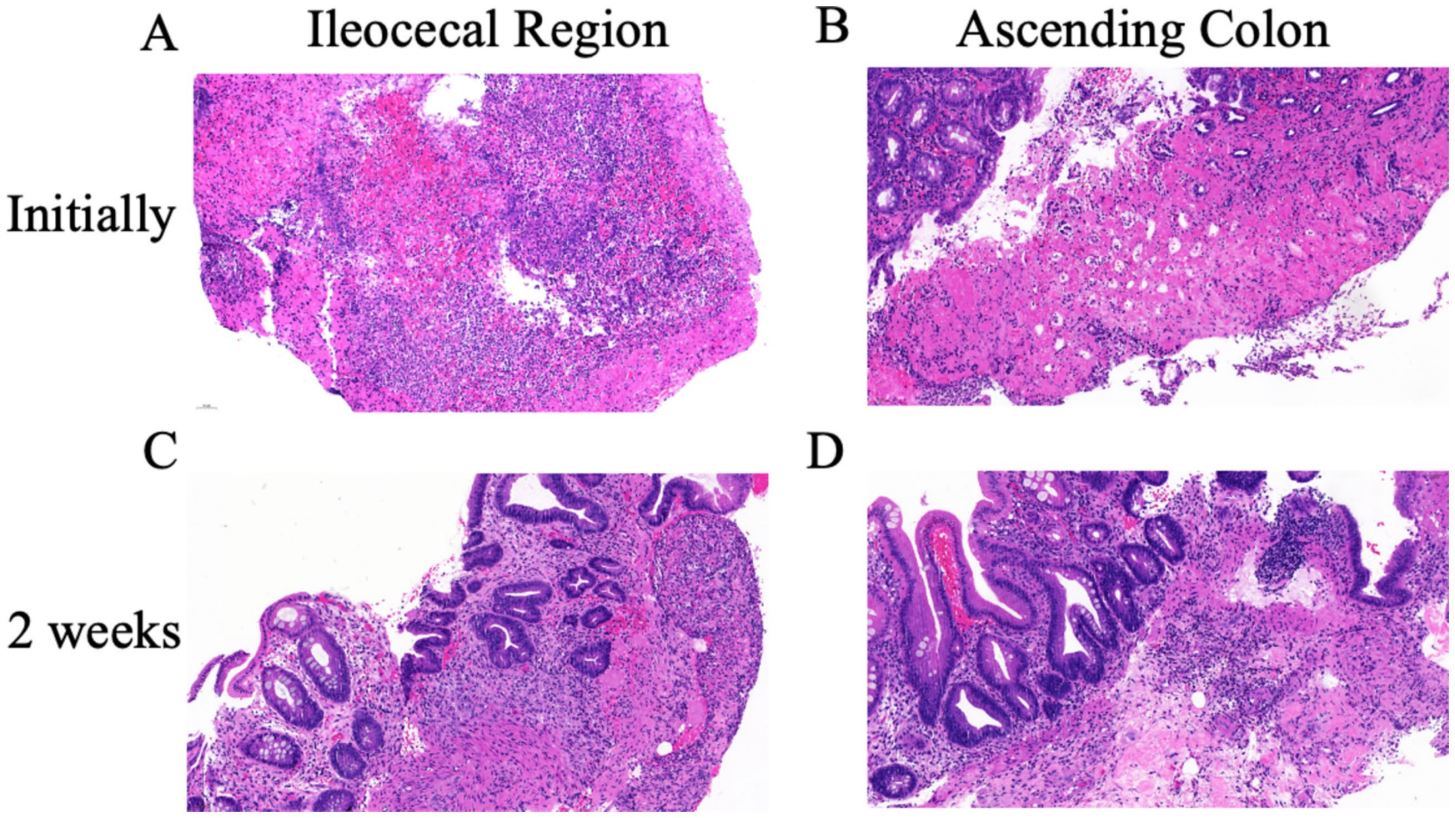
Pathological changes in the ileocecal and ascending colon lesions. **(A)** Initial pathological examination of ileocecal area revealed mucosal necrosis, hemorrhage and inflammation. **(B)** The ascending colon showed similar ischemic lesions with loss of surface and upper crypt epithelium, and lack of significant inflammation. After 2 weeks of treatment both the ileocecal valve **(C)** and the ascending colon **(D)** showed similar changes: chronic inflammation, focal erosion, crypt distortion and regenerative changes of crypt epithelium. No evidence of malignancy.

Upon admission, the abdominal examination revealed a soft abdomen, with marked discomfort upon palpation, but no significant tenderness or rebound tenderness. The assessment of the heart, lungs, and other physical systems demonstrated no abnormalities. Given the CT findings suggesting the potential presence of colon tumors, a comprehensive gastrointestinal endoscopy was performed. Colonoscopy revealed a tumor like lesion in the ileocecal region (Figure 2A), approximately 2–3 cm in diameter, with a spherical, convex appearance protruding into the intestinal lumen. The surface of the lesion showed congestion and erosion, with multiple yellow-white inflammatory secretions, and biopsy forceps displayed flexibility upon contact. The lesion’s margins were indistinct, with pronounced swelling and erosion. Additionally, patchy erosion was observed near the ileocecal area of the ascending colon (Figure 2B), with unclear boundaries and a slightly elevated surface where shallow ulcers had formed. Pathological examination of biopsy tissues from the ileocecal region (Figure 3A) and ascending colon (Figure 3B) revealed superficial bleeding, mucosal necrosis, fibrinous exudation, cellular infiltration of the lamina propria, and glandular loss and atrophy. These pathological features were consistent with ischemic enteritis. A comprehensive assessment based on clinical and endoscopic findings was recommended. The treatment regimen included ornidazole and ceftazidime for infection control, alprostadil injections to promote vasodilation, fluid resuscitation, bowel rest and oral enteric-coated lactic acid bacteria capsules to regulate intestinal flora.

After 2 weeks of treatment, the patient reported resolution of abdominal pain and began passing yellow, unformed stools. Follow-up colonoscopy showed that the previously visible mass in the ileocecal region (Figure 2C) had transformed into a depressed ulcer, approximately 2.5 cm by 1.8 cm, with a white fur-like surface. No active bleeding was detected, though the surrounding mucosa was congested and edematous. The original ulcer in the ascending colon had progressed to a shallow erosion (Figure 2D). Biopsies from these areas (Figures 3C,D) confirmed superficial bleeding, mucosal necrosis, fibrinous exudation, cellular infiltration, and glandular loss, consistent with ischemic enteritis. A follow-up examination was recommended post-treatment.

One month later, a follow-up colonoscopy showed the entire colon had returned to normal (Figures 2E,F), with no ulcers, erosions, or lesions. The patient reported no symptoms of discomfort. Follow-up after 6 months indicated no recurrence.

CT scans of the abdomen indicated significant thickening in the lower segment of the ascending colon and the ileocecal region, with intestinal shadows observed within. Enhanced imaging revealed multiple ring signs, and a slightly enhanced nodular shadow measuring approximately 24 mm was identified within the intestinal tract. Additionally, the surrounding fat gaps exhibited vague oozing.

## Discussion

Ischemic colitis (IC) is a condition that occurs when blood flow to the colon is reduced to a level insufficient to sustain cellular metabolic function, resulting in mucosal ulceration, inflammation, and hemorrhage (4). The incidence of IC ranges from 4.5 to 44 cases per 100,000 person-years (5–7). Prognosis largely depends on the time interval between symptom onset and diagnosis, as well as the presence of comorbidities (8). Key risk factors include age over 60, atrial fibrillation, smoking, obesity, and a history of venous thromboembolism (2). IC presents with diverse clinical manifestations, commonly including abdominal pain, diarrhea, and bloody stools. According to the different mechanisms of disease occurrence, IC can be divided into two categories: obstructive and non-occlusive (9). Obstructive ischemia arises from arterial or venous blockages, such as embolism or thrombosis, which lead to reduced blood flow and can potentially cause bowel necrosis. In contrast, non-occlusive ischemia results from systemic hypoperfusion, as seen in conditions like shock or heart failure, primarily affecting the watershed areas of the colon and resulting in ischemia. The primary distinction between these two types lies in their underlying mechanisms: obstructive ischemia is characterized by direct blockage of blood vessels, whereas non-occlusive ischemia is attributed to inadequate blood flow. Treatment strategies for obstructive ischemia often necessitate surgical intervention, while the management of non-occlusive ischemia typically emphasizes the improvement of systemic circulation to restore adequate blood flow. The severity and extent of ischemia can cause symptoms ranging from mild discomfort to acute abdominal pain and shock. Accurate diagnosis and treatment require a comprehensive evaluation of the patient’s symptoms, signs, and medical history. A thorough understanding of these features can facilitate early identification and intervention, improving patient outcomes.

Diagnosing IC often involves delays and uncertainties due to its symptoms closely resembling those of other abdominal conditions, such as pancreatitis, acute diverticulitis, small intestinal obstruction, and acute cholecystitis. Various diagnostic methods are available for IC, including CT scans (10), endoscopic examinations, surgical intervention, and histological analysis. Auxiliary examinations are crucial for diagnosing IC, identifying potential underlying causes, aiding in differential diagnosis, assessing the condition’s severity, and guiding treatment strategies.

CT scans with intravenous contrast agents are recommended as the preferred imaging modality for evaluating the distribution and stage of colitis in patients with suspected IC, offering good accuracy (11). Common CT findings indicative of IC include intestinal dilation and thickening, reduced or absent visceral enhancement, intestinal wall gas accumulation, presence of mesenteric or portal vein gas, and arterial or venous thrombosis (12). However, some patients with mild ischemia may not exhibit specific manifestations. In response to this challenge, radiologists have made considerable advancements in interpreting abdominal CT scans to improve diagnostic accuracy, particularly in cases of non-occlusive mesenteric ischemia (13). However, CT’s role in diagnosing certain cases of IC is limited (14–16). Colonoscopy is the gold standard for diagnosing IC (17) because it allows direct observation of changes in the intestinal mucosa, confirming the specific location and extent of lesions. It also enables tissue sample collection through biopsy for further pathological analysis, which is vital for differentiating IC from other conditions. Additionally, colonoscopy helps evaluate lesion severity and informs treatment plan development (18, 19).

Endoscopic findings of transient IC include petechial hemorrhages, fragile and edematous mucosa, segmental erythema, scattered erosions, longitudinal ulcerations, and sharply defined areas of involvement. Strictures associated with IC show full-thickness mucosal changes, lumen narrowing, and affected haustrations. In gangrenous colitis, endoscopic evaluation reveals mucosal features such as cyanosis and pseudopolyps. Clinicopathological characteristics include mucosal inflammation with erosion, granulation tissue, glandular atrophy, lamina propria hemorrhage, and hemosiderin-laden macrophages in the submucosa.

The primary goal in treating IC is to restore intestinal perfusion before irreversible damage occurs. Treatment generally includes etiological therapy, supportive therapy, and surgical intervention (20). Etiological therapy focuses on enhancing blood flow and may involve addressing thrombotic issues through anticoagulant and thrombolytic treatments. Supportive therapy includes fluid resuscitation, management of electrolyte imbalances, and administration of antibiotics to prevent secondary infections (21). In severe or irreversible ischemia, surgical intervention, such as necrotic bowel resection, may be necessary (22). Postoperative management involves providing nutritional support and monitoring for complications, with the aim of promoting recovery and minimizing the risk of recurrence.

The prognosis of IC is influenced by several factors, including the extent of ischemia, the patient’s overall health, and the timeliness of treatment. Notably, the prognosis varies depending on the affected side, with left-sided ischemic colitis generally exhibiting a better prognosis than right-sided ischemic colitis. While some patients achieve favorable outcomes through conservative and supportive care, severe cases may have a poor prognosis. Additionally, postoperative complications such as intestinal fistula, infection, and ischemia can adversely affect outcomes (23). Regarding the potential sequelae of patients with IC, there is currently no evidence to suggest a progression to chronic colitis (24). Only a limited number of cases have documented the occurrence of colon stenosis following IC (25), indicating that further research is necessary to establish a definitive correlation between these two conditions.

Early diagnosis and timely intervention are crucial for improving prognosis. Currently, there is a lack of established diagnostic biomarkers for IC. Blood tests commonly use lactic acid, C-reactive protein, white blood cell counts, D-dimer, phosphate, and creatine kinase (CK) as auxiliary diagnostic tools. Several novel indicators have been identified as being associated with IC (26), including (27) ischemic modified albumin (IMA), and the major urinary metabolite of prostaglandin E2 (PGE-MUM) (28). However, further research is needed to validate their accuracy (29). This underscores the necessity of promoting early diagnosis of ischemic colitis from a multidisciplinary perspective (27, 30).

A notable characteristic of this patient with IC is the endoscopic appearance resembling a colon mass, which is relatively uncommon and can easily lead to misdiagnosis. In the present case, local ischemia in the ascending colon could be secondary to a mechanism of distal embolism, possibly due to atrial fibrillation, affecting a branch of the superior mesenteric artery supplying the right colon. However, this speculation requires further research evidence for validation. The fact that the recto-sigmoid was normal is an argument against the diagnosis of non-occlusive mesenteric ischemia. A literature review revealed only a limited number of similar reports (31, 32), with some cases only diagnosed as IC after intestinal resection (33). There is a significant gap in mechanistic studies that investigate the underlying causes of this phenomenon. Consequently, pathologists and gastroenterologists should remain vigilant regarding this atypical presentation to avoid unnecessary surgical interventions. This study has several limitations. Some biomarkers associated with IC could not be assessed at our institution, and there is a lack of comparative data on these indicators before and after treatment. Additionally, this review includes some small-scale studies, such as case reports. Larger clinical studies are needed to elucidate the mechanisms and prevalence of colonic masses in IC, deepening our understanding of this rare endoscopic manifestation.

## Conclusion

In summary, IC is a complex condition that presents with nonspecific symptoms and lacks well-developed diagnostic biomarkers, making it difficult to distinguish from other abdominal diseases and complicating early diagnosis (34). Traditional imaging techniques may not promptly detect early ischemic changes, and while endoscopic methods are effective, they can be invasive. Atypical findings may require pathological confirmation, potentially delaying diagnosis. Additionally, the diverse causes of IC result in a wide range of clinical presentations, necessitating personalized treatment strategies. These factors complicate the diagnosis and management of IC. This complexity underscores the importance of understanding and recognizing IC’s endoscopic features to ensure accurate and timely diagnosis and treatment, thereby avoiding misdiagnoses and delays. There is a pressing need to raise awareness about IC and improve diagnostic and management guidelines. Future research should focus on fostering interdisciplinary collaboration among internal medicine, surgery, radiology, and pathology to enhance diagnostic and therapeutic strategies, ultimately improving patient outcomes.

## Data Availability

The original contributions presented in the study are included in the article/supplementary material, further inquiries can be directed to the corresponding author.
